# Ex Vivo Buccal Permeability of Nanostructured Lipid Carriers (NLCs) Associated with a Peptide Drug Model

**DOI:** 10.3390/pharmaceutics18040416

**Published:** 2026-03-29

**Authors:** Sebastián Vargas-Valderrama, Javier O. Morales

**Affiliations:** 1 Drug Delivery Lab, Department of Pharmaceutic Science and Technology, Faculty of Chemical and Pharmaceutical Sciences, University of Chile, Santiago 8380494, Chile; sebastian.vargas.1@ug.uchile.cl; 2 Advanced Center for Chronic Diseases, ACCDiS, Santiago 8380494, Chile; 3 Center of New Drugs for Hypertension and Heart Failure (CENDHY), Santiago 8380494, Chile; 4 Nanocarrier Advanced Inhalation Research Center, NanoAIR, Santiago 8380494, Chile

**Keywords:** nanostructured lipid carriers, buccal permeability, ex vivo, Franz diffusion cells, porcine buccal mucosa, angiotensin II, peptide delivery

## Abstract

**Background/Objective:** Buccal delivery offers a potential route to circumvent gastrointestinal degradation and hepatic first-pass metabolism, but hydrophilic peptides typically exhibit limited mucosal permeation. Nanostructured lipid carriers (NLCs) have been proposed as delivery platforms capable of modulating interfacial interactions and improving mucosal transport. This study aimed to quantitatively evaluate the ex vivo buccal permeation of angiotensin II (Ang II), used as a hydrophilic peptide model, when associated with NLCs compared with free peptide under matched Franz diffusion cell conditions. **Methods:** Ang II-associated NLCs were prepared by melt emulsification combined with a low-energy injection technique. Particle size, polydispersity index, and zeta potential were determined by dynamic light scattering and laser Doppler electrophoresis. Association efficiency and drug loading were quantified by indirect spectrofluorometric analysis. Ex vivo permeation studies were conducted using porcine buccal mucosa mounted in Franz diffusion cells, and cumulative permeation, steady-state flux, and apparent permeability coefficients were calculated. **Results:** The NLCs exhibited nanometric size, moderate polydispersity, and association efficiency above 80%, and remained colloidally stable at 4 °C for 28 days. In ex vivo experiments, Ang II-associated NLCs showed measurable cumulative permeation, reaching approximately 9% after 2 h, whereas free Ang II was not detected in the receptor compartment under the tested conditions. **Conclusions:** This work provides a quantitative ex vivo buccal transport comparison of a hydrophilic peptide model delivered as NLC-associated versus free peptide under matched Franz cell conditions. The findings support further investigation of NLC-based approaches for buccal delivery of vasoactive peptides and provide a rational basis for future in vivo evaluation of mucosal delivery performance and systemic exposure.

## 1. Introduction

Hypertension is defined as a persistent elevation of arterial blood pressure above 140/90 mmHg. In 2023, the World Health Organization reported a global prevalence of 33.1% among adults aged 30–79, with two-thirds of cases occurring in low- and middle-income countries [1]. As a major risk factor for cardiovascular disease (a leading cause of mortality worldwide), poorly controlled hypertension contributes to multiorgan dysfunction affecting the heart, kidneys, brain, and vasculature [2,3]. Although several pharmacological classes are clinically established for hypertension management, their long-term performance is often compromised by adverse events, suboptimal adherence, and cumulative toxicity [3,4,5,6,7,8]. These limitations underscore the need for alternative therapeutic strategies.

The renin–angiotensin system (RAS) plays a central role in cardiovascular regulation. Classical RAS signaling through the angiotensin II type-1 receptor (AT1R) promotes vasoconstriction and cardiac hypertrophy, whereas counter-regulatory pathways, mediated partly by angiotensin (1–9) (H_2_N-Asp-Arg-Val-Tyr-Ile-His-Pro-Phe-His-OH) via AT2R, exert vasodilatory and cardioprotective effects [9,10,11,12,13,14,15]. Peptide-based therapeutics targeting these pathways show promising biological activity; however, their clinical translation is restricted by low oral bioavailability, rapid degradation, and limited epithelial permeability [16,17,18]. For the purposes of this proof-of-concept ex vivo study, angiotensin II (Ang II) (H_2_N-Asp-Arg-Val-Tyr-Ile-His-Pro-Phe-OH) was selected as a hydrophilic peptide model based on its analytical tractability and relevance to the renin–angiotensin system.

Nanotechnology-based carriers have emerged as a viable strategy to overcome peptide delivery barriers. Lipid nanoparticles provide protection against enzymatic degradation, improve solubility, and allow modulation of release kinetics. At present, various types of nanoparticles [19,20,21] have been developed, among which nanostructured lipid carriers (NLCs), composed of a blend of solid and liquid lipids, offer enhanced association efficiency, stability, and loading capacity relative to first-generation systems and have demonstrated compatibility with hydrophilic bioactives [22,23,24]. Several methods for formulating NLCs with peptides [25] and proteins [26] incorporated into their structure have been described, often relying on complex synthesis techniques such as ion-pair formation. In contrast, the present study proposes an alternative approach based on peptide association through passive adsorption. Moreover, it addresses a largely unexplored area: the buccal permeation of peptide-associated NLCs.

The buccal route is an attractive alternative for systemic peptide delivery because it avoids first-pass hepatic metabolism and reduces gastrointestinal degradation. The buccal mucosa features a non-keratinized, highly vascularized epithelium with a surface area of approximately 50 cm^2^ and rapid cellular turnover, characteristics that support transmucosal drug absorption [27,28]. Porcine buccal mucosa is widely accepted as a surrogate model due to its morphological and permeability similarities to human tissue [29].

## 2. Materials and Methods

### 2.1. Materials

Gelucire^®^ 44/14 was kindly provided by Gattefossé (Saint-Priest, France). Tween^®^ 80, Mygliol^®^ 812, human angiotensin II (MW = 1046.18 g/mol), and dialysis membranes (MWCO 7000 Da) were purchased from Sigma-Aldrich (St. Louis, MO, USA). Potassium dihydrogen phosphate (KH_2_PO_4_), sodium hydroxide (NaOH), ethanol, hydrochloric acid, and Amicon^®^ Ultra-15 centrifugal filter units (MWCO 10 kDa) were obtained from Merck Millipore (Darmstadt, Germany). Tissue scissors and carbon steel blades for tissue slicing were obtained from Fisher Scientific (Waltham, MA, USA).

### 2.2. Preparation and Characterization of Nanostructured Lipid Carriers (NLCs) Loaded with Ang II

#### 2.2.1. Synthesis of NLCs Loaded with Ang II

NLCs were prepared following the methodology previously described by Zhang et al. [30], with modifications introduced by Ortiz et al. [31]. The formulation consisted of 600 mg of Gelucire^®^ 44/14, 150 mg of Miglyol^®^ 812, and 300 mg of Tween^®^ 80. The nanoparticles were produced using a melt emulsification method combined with a low-energy injection technique. The lipid phase was heated to 60 °C for 5 min, after which the solubilized peptide was added to 13.95 mL of preheated water at the same temperature. The aqueous phase was gradually added to the lipid phase under constant stirring. The resulting formulation was rapidly cooled at 4 °C for 30 min without further agitation.

#### 2.2.2. Physicochemical Characterization of NLCs

The polydispersity index (PDI) and hydrodynamic diameter (HD) were determined by dynamic light scattering (DLS) using a Zetasizer Nano ZS (Malvern Panalytical Ltd., Malvern, UK). Samples were diluted 1:10 with Milli-Q water and analyzed in triplicate. Zeta potential (ZP) was measured by laser Doppler electrophoresis using the same instrument.

#### 2.2.3. Association Efficiency and Drug Loading of Ang II

Following synthesis, a fraction of the formulation was transferred into Amicon^®^ Ultra centrifugal filter units (MWCO 10 kDa) and centrifuged at 4000 rpm for 20 min. The filtrate was analyzed by spectrofluorometry to quantify the amount of non-associated peptide. Calibration curves (r^2^ = 0.9997; LOD = 0.0197 ppm; LOQ = 0.0597 ppm) were constructed with Milli-Q water over a concentration range of 1.5–100 ppm with triplicate measurements. The excitation and emission wavelengths were set at 274 nm and 302 nm, respectively, with a slit width of 5 nm, based on the fluorescence spectrum of tyrosine reported in the literature [32]. Association efficiency (*AE*) (Equation (1)) and drug loading (DL) (Equation (2)) of Ang II were calculated using an indirect quantification method:

(1) $$AE\left(\%\right)=\frac{Initial\;amount\;of\;Ang\;II-Amount\;of\;non-entrapped\;Ang\;II}{Initial\;amount\;of\;Ang\;II}*100$$

(2) $$DL\left(\%\right)=\frac{Initial\;amount\;of\;Ang\;II-Amount\;of\;non-entrapped\;Ang\;II}{Total\;amount\;of\;solids}*100$$

The initial amount of peptide corresponds to the mass added during synthesis, whereas the free peptide refers to the non-entrapped fraction quantified in the filtrate.

#### 2.2.4. Stability Study of Nanostructured Lipid Carriers (NLCs)

The stability of NLC suspensions was assessed over 28 days post-synthesis at three storage temperatures (4 °C, 25 °C, and 37 °C) as a preliminary assessment of the physicochemical stability of the developed nanostructured lipid carriers (NLCs) [33]. Physicochemical parameters including hydrodynamic diameter, polydispersity index (PDI), and zeta potential were monitored using a Zetasizer Nano ZS. Both peptide-loaded and blank NLCs were evaluated. Two formulations from the same batch were divided into three separate containers and stored under the respective temperature conditions. For each measurement, 100 μL of the suspension was diluted in 900 μL of Milli-Q water and analyzed in triplicate.

### 2.3. Ex Vivo Buccal Permeability Study

#### 2.3.1. Collection of Porcine Buccal Mucosa

Porcine cheeks were obtained from a local slaughterhouse, transported in phosphate-buffered saline (PBS, pH 7.4) at 4 °C, and cryopreserved in 30% sucrose solution in PBS (pH 7.4) at −80 °C to preserve epithelial integrity [34].

#### 2.3.2. Mucosal Integrity and Ex Vivo Buccal Permeability Study of NLCs

The study design followed the methodology described by Kulkarni et al. [35]. Mucosal integrity was assessed using fluorescein isothiocyanate–dextran 20 kDa (FD-20) at 200 ppm. FD-20 permeation was quantified by spectrofluorometry at excitation and emission wavelengths of 498 nm and 518 nm, respectively, with a slit width of 5 nm. Calibration curves (r^2^ = 0.9999; LOD = 0.0742 ppm; LOQ = 0.225 ppm) were constructed in PBS (pH 7.4) over a concentration range of 0.01–12 ppm, with triplicate measurements.

Prior to use, cryopreserved tissue was rinsed with PBS (pH 7.4) to remove residual cryoprotectants. Buccal mucosa samples (~2 cm^2^) were dissected from pig cheeks, washed, and separated from underlying connective tissue and muscle to yield epithelial sheets of ~500 μm thickness using surgical scissors and carbon steel blades.

Permeability assays were conducted using Franz diffusion cells (Teledyne Hanson Research, Phoenix model, Chatsworth, CA, USA). The tissue was mounted with the epithelium facing the donor chamber. The receptor chamber was filled with 10 mL of PBS (pH 7.4) and maintained at a controlled temperature (37 °C) under continuous stirring (400 rpm). The donor chamber was loaded with 900 μL of NLC suspension (post-filtration) (corresponding to 850 µg/mL Ang II) and 100 μL of FD-20 solution. A free Ang II solution at the same concentration was used as the control. Samples of 400 μL were withdrawn from the receptor compartment at 9, 18, 27, 36, 45, 54, 63, 90, 102, and 120 min, with replacement by equal volumes of fresh buffer. Ang II quantification was performed by spectrofluorometry (λex = 274 nm, λem = 302 nm, slit width = 10 nm). Calibration curves of Ang II (r^2^ = 0.9985; LOD = 0.0742 ppm; LOQ = 0.225 ppm) were prepared in PBS (pH 7.4) at concentrations ranging from 5 to 100 ppm. All experiments were conducted using three independent replicates for each measurement.

The steady-state flux (*Jss*) and apparent permeability coefficient (*Papp*) were calculated according to Equations (3) and (4), where *K* is the slope of the cumulative amount of drug permeated versus time, *A* is the diffusion area of the buccal mucosa, and *C*_0_ is the initial concentration of Ang II in the donor compartment:

(3) $$Jss=\frac{K}{A}$$

(4) $$Papp=\frac{Jss}{C_{0}}$$

### 2.4. Statistical Analysis

All numerical data are expressed as mean ± standard deviation (SD). Comparisons between two groups were performed using Student’s t-test, while one-way ANOVA followed by Tukey’s post hoc test was applied for comparisons among three or more groups. Statistical analyses were carried out using GraphPad Prism 8, with significance defined at *p* < 0.05. For comparison of release profiles (Figure S1), the similarity factor (f_2_) was calculated using DDSolver software (version 1.0); values greater than 50 were considered indicative of profile similarity.

## 3. Results

### 3.1. Preparation and Characterization of Ang II-Loaded Nanostructured Lipid Carriers (NLCs)

#### 3.1.1. Physicochemical Characterization of Nanostructured Lipid Carriers (NLCs)

**Figure 1:**
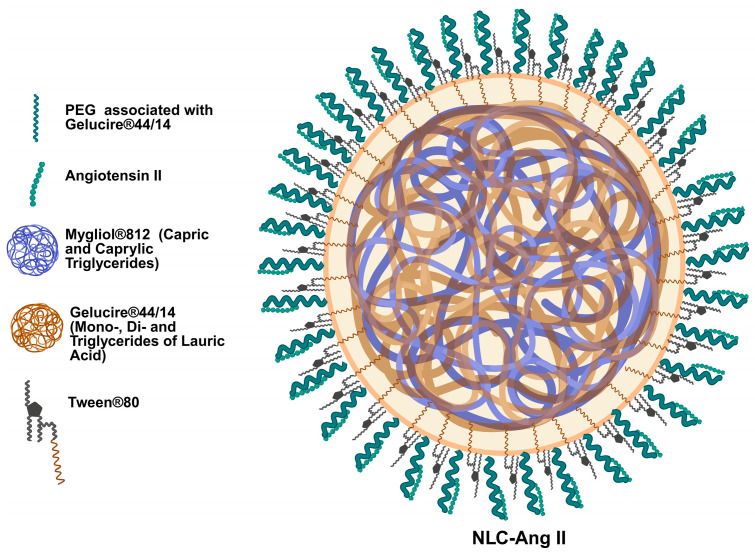
Illustrative schematic of the proposed organization of Ang II-associated NLCs based on formulation composition.

Significant differences were observed (Table 1) in hydrodynamic diameter (HD), with Ang II-loaded NLCs (Figure 1 presents an illustrative representation of the formulation composition employed) showing values of 83.73 ± 0.7 nm and 91.52 ± 0.19 nm compared to their unloaded counterpart (72.10 ± 8.91 nm). The polydispersity index (PDI) of both formulations displayed similar magnitudes, with narrow size distributions for NLC-Ang II (0.219 ± 0.010), NLC-Ang IIc (Nanostructured lipid carrier associated with concentrated Ang II) (0.263 ± 0.009) and empty NLCs (0.261 ± 0.016). Both zeta potentials (ZPs) were slightly negative, with Ang II-loaded NLCs tending toward greater neutrality (−3.74 ± 0.40 mV and −2.89 ± 0.42 mV) compared with unloaded NLCs (−6.20 ± 1.10 mV). Regarding association efficiency (AE), this nanosystem exhibited a high peptide association (87.29 ± 0.34% for NLC-Ang II and 83.61 ± 0.06 for NLC-Ang IIc) with a drug loading (DL) of 0.484 ± 0.003% for NLC-Ang II and 0.907 ± 0.001 for NLC-Ang IIc.

#### 3.1.2. Stability Study of Nanostructured Lipid Carriers (NLCs)

**Figure 2:**
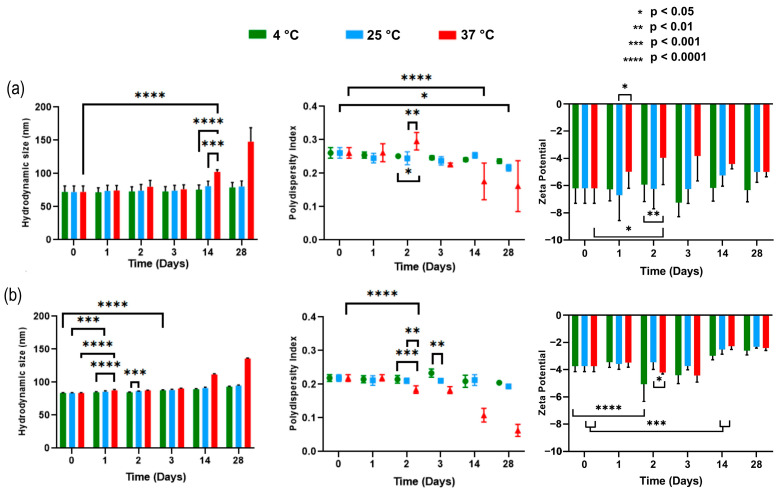
Stability at 28 days of empty nanostructured lipid carriers (NLCs) (**a**) and associated with Angiotensin II (NLC-Ang II) (**b**) exposed to 4, 25, and 37 °C. The asterisks indicate significant differences between compared groups where the greatest significance is marked according to *p*-value (*: <0.05; **: <0.01; ***: <0.001; ****: <0.0001).

Following formulation, the nanoparticles were stored at different temperatures (4 °C, 25 °C, and 37 °C), and HD (Table 2), PDI, and ZP were monitored over time (Figure 2).

Empty NLCs did not exhibit significant changes in HD during the 28-day evaluation period when stored at 4 °C and 25 °C. However, peptide-loaded NLCs showed greater sensitivity to storage conditions: at 4 °C, significant HD variations appeared after day 3, while at 25 °C these changes were already evident on day 1. At 37 °C, both formulations displayed the most pronounced variations, with rapid and marked increases in HD.

For PdI, both empty and loaded NLCs remained below 0.3 at 4 °C and 25 °C throughout the study, indicating acceptable homogeneity in particle size distribution.

As described previously, most nanoparticles exhibited slightly negative ZP values upon synthesis. These potentials remained stable over time, regardless of storage temperature.

### 3.2. Ex Vivo Buccal Permeation of Nanostructured Lipid Carriers (NLCs)

Permeability studies were conducted after thawing and washing porcine buccal tissue obtained from the slaughterhouse using the NLC formulation synthesized with 11.6 mg of Ang II (NLC-Ang IIc). Sections were prepared and mounted in Franz diffusion cells for triplicate assays. FD-20 (200 ppm) was co-incubated with the test samples (free Ang II solution and Ang II-loaded NLC) to monitor membrane viability throughout the experiment.

**Figure 3:**
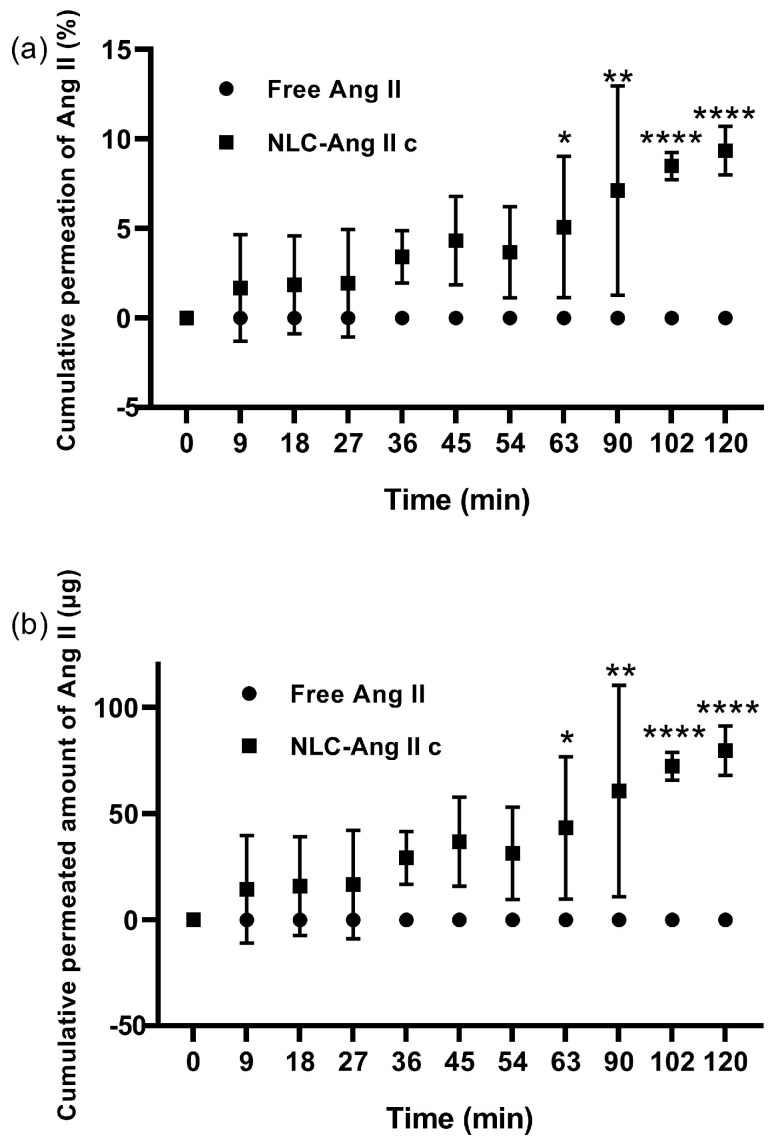
Cumulative ex vivo permeation profiles of angiotensin II (Ang II) in solution and loaded in nanostructured lipid carriers (NLC-Ang IIc) through excised porcine buccal epithelium, with PBS (pH 7.4), expressed as percentage (**a**) and cumulative amount (**b**). The asterisks indicate significant differences between compared groups where the greatest significance is marked according to *p*-value (*: <0.05; **: <0.01; ****: <0.0001).

Permeation profiles (Figure 3a) demonstrated significantly higher diffusion for Ang II-loaded NLCs (9.35 ± 1.36%) after 2 h without reaching the plateau, whereas free Ang II solution remained below the quantification limit in the receptor compartment under the tested conditions. Both studies showed significant differences starting at 63 min. The apparent permeability coefficient (Papp) of the loaded NLCs was 1.93 ± 0.11 (×10^−5^) cm/s, with a steady-state flux (Jss) of 1.65 ± 0.09 (×10^−2^) µg/cm^2^·s.

## 4. Discussion

### 4.1. Preparation and Characterization of Nanostructured Lipid Carriers (NLCs) Loaded with Ang II

#### 4.1.1. Physicochemical Characterization of Nanostructured Lipid Carriers (NLCs)

The observed enlargement in hydrodynamic diameter upon peptide association is plausibly mediated by the binding of angiotensin II to surface-exposed PEGylated chains on the nanoparticles, which promotes an effective increase in the hydrodynamic boundary layer. Previous work from our group using the same Gelucire^®^ 44/14/Miglyol^®^ 812/Tween^®^ 80 platform prepared by a similar low-energy method reported spherical nanoparticles by TEM [31,36]. However, no TEM characterization was performed in the present study. Therefore, the structural organization of the Ang II-associated NLCs discussed here is supported by indirect physicochemical characterization, although it was not confirmed by direct morphological imaging in the present study.

The polydispersity index (PDI) is a parameter that reflects the uniformity of particle size within a formulation and can take values ranging from 0 to 1. The closer this value is to zero, the greater the homogeneity of the sample; conversely, values close to one indicate a more heterogeneous size distribution. In this study, both empty NLCs and Ang II–loaded NLCs presented polydispersity index (PdI) values below 0.3 [30,37]. This indicates that the formulation exhibits a sufficiently homogeneous size distribution to be considered optimal, which is characteristic of monodisperse systems.

Regarding the zeta potential (ZP), the values obtained were slightly negative for the formulated nanoparticles. This negative charge can be associated with the presence of surfactants and free fatty acids on the surface, especially those derived from the hydrolysis of glycerides from Gelucire^®^ 44/14 [38]. Upon association of the nanoparticles with Ang II, the magnitude of the ZP decreased in terms of its negative value. This decrease in zeta potential may be due to the charge of the peptide; using the Protein Calculator v3.4 tool for peptides and proteins [39], an isoelectric point of pH 7.21 was obtained. It can therefore be estimated that Ang II, at the acidic pH (pH = 5.5) resulting from the use of Milli-Q water, was positively charged, acquiring a net charge of +1, conferred by the presence of basic amino acids such as arginine and histidine. This would allow the peptide, when interacting with surface constituents, to reduce the negative zeta potential of the nanoparticle.

Zeta potential is of particular importance in the study of emulsion and colloidal stability since, according to DLVO electrostatic theory, such stability is explained as a function of the interactions between the surfaces of particles suspended in a medium, considering the total interaction energy generated between them [40]. In terms of electrostatic stability, a ZP greater than ±30 mV is considered indicative of good particle repulsion and therefore greater stability, whereas values below ±30 mV, such as those observed in this study, could favor aggregation and system instability. It should be noted that nanoparticles with values between −10 mV and +10 mV can be classified as neutral [41]. This neutrality could confer a certain advantage to these nanosystems, as they may be able to avoid macrophage uptake and thus increase circulation time in the bloodstream, in addition to avoiding cytotoxicity that may occur in certain nanosystems with excessive positive charge density [42].

Despite this apparent electrostatic instability, the nanoparticles could maintain their stability through an alternative mechanism known as steric stabilization, which is conferred by polymeric compounds and was later incorporated into the extended DLVO theory [40]. The components responsible for this property in the nanoparticles are the PEGylated chains present on their surface, provided by Gelucire^®^ 44/14 and the non-ionic surfactant used as stabilizer (Tween^®^ 80). This non-ionic surfactant, having a shorter PEGylated chain (20 repeating ethylene oxide units), can provide these nanoparticles with a close-contact solvation barrier, while the longer PEGylated chains of Gelucire^®^ 44/14 (33 repeating ethylene oxide units) are able to extend further into the aqueous medium, generating a hydrated cloud with excluded volume that prevents particle–particle contact through steric repulsion [43,44].

Although both formulations exhibited DL values below 1%, the consistently high AE, even after doubling the initial peptide amount, suggests that the system can associate the available peptide under the tested conditions.

#### 4.1.2. Stability Study of Nanostructured Lipid Carriers (NLCs)

The kinetic stability study allows the behavior of colloidal systems in aqueous solution to be described over time [45]. For this reason, different storage temperatures were tested. The behavior observed at 37 °C can be attributed to the increase in the kinetic energy of the nanoparticles caused by temperature, favoring a greater number of collisions and interactions between them, promoting aggregation processes and, consequently, an increase in their hydrodynamic diameter.

In addition, it should be considered that Ortiz et al. [31], through differential scanning calorimetry (DSC) studies, evaluated the physical state of the components in NLC formulations. Their results showed that Gelucire^®^ 44/14, with a theoretical melting point of 44 °C, experienced a shift toward 38 °C when it was part of the nanoparticle. In contrast, when the components were only mixed (without nanoparticle structuring), the melting point decreased only to 40 °C. This behavior was attributed to the interaction between Gelucire^®^ 44/14, Miglyol^®^ 812, and Tween^®^ 80, which could facilitate partial solubilization of solid lipids, favoring earlier melting. The additional decrease in melting point in the case of the structured nanoparticle could be explained by the close interaction between components during the emulsification process, in which they are in intimate contact at elevated temperatures and subsequently stabilize upon rapid cooling, generating a compact and homogeneous structure.

In this context, considering that the melting point of Gelucire^®^ 44/14 is close to the storage temperature of 37 °C, it is plausible to assume that the nanoparticles begin to undergo a partial melting process, leading to an expansion of the colloidal system due to a decrease in cohesion between its constituents [31].

As expressed in the previous section, most nanoparticles exhibited a slightly negative zeta potential at the time of synthesis. Due to its low magnitude, this potential can be characterized as practically neutral. Throughout the study, it was observed that these potentials remained stable over time, regardless of storage temperature.

### 4.2. Ex Vivo Buccal Permeation of Nanostructured Lipid Carriers (NLCs)

For the present permeability study, it was considered that these systems may induce cellular damage; therefore, it was important to evaluate membrane integrity using an integrity marker [46], which allows verification of whether the buccal mucosa tissue suffered damage during extraction, transport, preservation, or during permeability studies [46,47]. This ensures that the results obtained are not a consequence of compromised tissue integrity. One integrity marker studied is fluorescein isothiocyanate-labeled dextran (FITC-dextran), with a molecular weight of 20,000 Da (FD-20), which is highly hydrophilic. If this marker were to permeate, it would indicate a loss of integrity of the paracellular pathway (between cells), since FITC-dextran of such molecular weight is incapable of diffusing through the buccal mucosa [35,48,49,50,51]. Kulkarni et al. [35] postulated that the passage of approximately 0.1% of the marker after 4 h of study may be indicative of loss of tissue integrity. A dedicated cytocompatibility assessment in buccal cell models was not included in the present study. Therefore, although tissue integrity was monitored during the ex vivo assay using FD-20, these data should not be interpreted as a standalone safety evaluation of the formulation.

The 2 h duration was selected to balance physiological relevance and preservation of ex vivo tissue integrity. Buccal dosage systems are exposed to salivary clearance and mechanical disturbance [19,27,28], while prolonged ex vivo experiments may progressively alter barrier properties [35]. Within this timeframe, the present study captured clear discrimination between free Ang II and NLC-associated Ang II and enabled calculation of Jss and Papp under controlled conditions.

NLC-Ang IIc denotes the formulation prepared with the higher Ang II input (11.6 mg). This formulation was selected for permeability studies because it provided the higher DL and therefore the greatest peptide amount per administered donor volume. Short-term stability was evaluated only for blank NLC and NLC-Ang II as representative formulations; however, the absence of an independent stability evaluation for NLC-Ang IIc represents a limitation of the present study.

During the study, FD-20 was used together with the sample under study (Ang II in solution, NLC–Ang IIc) to verify membrane viability over time.

The absence of permeation of free Ang II through the buccal mucosa is expected due to the nature of this tissue. It has been described that the route through which biological products and hydrophilic molecules permeate is the paracellular or intercellular pathway (between cells). This logic is based on the existence of membrane-coating granules in the buccal epithelium, which can secrete an amorphous material into the intercellular space (with occasionally short stacks of lipid lamellae). The secreted lipid content is polar in nature, composed of glycosylceramides [52], which confers greater affinity of this pathway for the transit of this type of molecule. However, although the intercellular space is more favorable for this type of molecule, the paracellular pathway may be influenced by the extruded lipid material, as this material could restrict the movement of large molecules [53,54]. Therefore, this could generate some impediment to the passage of peptides such as Ang II.

Although the measured DL values were below 1%, the formulation with the higher loading still enabled measurable ex vivo transport at the donor concentration used in this study. These data support proof-of-concept buccal delivery, but they do not by themselves establish clinical dose feasibility, which will depend on therapeutic dose requirements and further formulation optimization. The increase in active compound permeation when associated with NLCs can be explained by the use of permeation enhancers in the NLC formulation. These are substances capable of increasing the permeation rate of a co-administered drug across a biological membrane, and they can act through different mechanisms depending on the agent used [54]: increased drug distribution within the tissue, extraction of intercellular lipids, interaction with epithelial protein domains, and increased drug retention on the buccal mucosal surface.

In this case, NLCs were synthesized using Tween^®^ 80, a polysorbate with surfactant capacity, which has been described as having permeation-enhancing effects due to perturbation of protein domain integrity and extraction of intercellular lipids [54,55,56]. Its solubilizing effect on buccal epithelial membrane lipids allows greater membrane fluidization, improving permeability through the paracellular route. However, it has also been indicated that if surfactant concentration is high, it could affect the transcellular route, since extraction of lipids from the cellular membrane could facilitate improved transcellular transport. Therefore, solubilization of both intercellular and epithelial lipids could exert a permeation-enhancing effect on both transport pathways [54].

Another component that could influence the permeation-enhancing effect is one of the constituents of Gelucire^®^ 44/14, such as lauric acid and caprylic acid [57]. It has been suggested that, due to their presence in the membrane, these compounds could alter interactions between hydrocarbon chains, affecting lipid packing order in buccal epithelial cell membranes, thereby making their structure less organized and favoring passage through the transcellular route. Another possible mechanism is fluidization of intercellular lipids, which would make the membrane more permeable to substances, thus improving peptide diffusion via the paracellular route [54,58].

Additionally, PEG associated with the surface of these nanoparticles could further benefit permeation, since it has been shown to confer improved diffusion across mucosal surfaces [44], allowing the nanoparticle to move more freely in this environment and facilitating its transit toward epithelial cells.

It should be mentioned that the high variability of permeability results may be due to the intrinsic variability associated with the use of excised ex vivo porcine cheeks in permeation experiments, rather than well-controlled cell culture models, although the latter are less physiologically representative [48]. Nevertheless, this behavior is consistent with what has been reported in other studies conducted in the same tissue [34,43], which supports the reliability of the results obtained.

In a study conducted by Teubl et al. [59], the influence of size on polymeric nanoparticles was demonstrated. In this study, uptake of neutral polystyrene nanoparticles was shown to depend on size, with 200 nm particles penetrating deeper regions of the mucosa compared to 25 and 50 nm nanoparticles. This behavior was attributed to the structure of the buccal mucosa, particularly the mucus layer and microplicae. The first barrier to overcome is mucus, composed of mucin. To penetrate mucus, nanoparticles must avoid adhesion to mucin fibers and/or overcome filtration and size-exclusion effects. It was described that uncharged nanoparticles cannot adhere to mucin fibers; however, due to their size being less than 100 nm, they may be affected by a small pore known as a “pocket,” causing these nanoparticles to become trapped and hindering their penetration into the mucosal structure, thus reducing diffusion rate.

Another consideration is microplicae, which are ridge-shaped folds located on the buccal epithelial surface. These microfolds have diameters ranging from 200 to 500 nm. Small nanoparticles can become trapped in these folds, making them less susceptible to cellular uptake due to the greater energy required for the membrane to envelop them. If many nanoparticles accumulate in these spaces, electrostatic repulsion effects between particles may be generated, limiting close and sustained contact between particles and the cell membrane. Therefore, despite their small size (which would initially facilitate epithelial penetration), these particles may exhibit reduced cellular uptake efficiency by becoming transiently retained within the folds without achieving effective interaction with active entry sites. It should also be noted that the present study quantified Ang II recovery in the receptor compartment as the primary permeability endpoint and did not establish a complete mass balance, including residual donor content or tissue-associated peptide. Therefore, the gap between the rapid in vitro release profile and the lower receptor recovery after 2 h should be interpreted as reflecting the barrier function of the buccal epithelium [59], together with possible tissue retention [60,61] and/or peptide loss during the ex vivo assay, rather than as a direct measure of total peptide disposition.

Compared with previous studies employing systems such as SEDDSs (Self-Emulsifying Drug Delivery Systems) [62,63,64,65], liposomes [66,67,68,69], polymeric micelles [70], and others NLCs [71,72,73], the present formulation showed Papp values that compare favorably with several literature examples. Direct cross-study comparison should be interpreted cautiously because reported Papp values depend on tissue source, donor concentration, experimental configuration, and analytical methodology. Within these limitations, the observed permeation performance is consistent with a permeation-enhancing contribution from the formulation components previously described. It is noteworthy that the development of this nanoparticle did not require more complex techniques to achieve high association efficiency of the biological material within its structure, in contrast to what is commonly necessary for liposomes and SEDDSs. Liposomal formulations [74] often rely on active loading strategies, such as mass transfer or electrostatic attraction, whereas SEDDSs [75] typically demand longer processes, including hydrophobic ion pairing, chemical modification, or double emulsification (a technique also applied in the production of NLCs [76]).

The formulation developed here showed lower values than those reported by Wai et al. [43,77] in their study on lipid-core micelles (LCMs). This difference could be explained by the lipophilicity of the permeation enhancer used there, Span^®^80 (HLB: 4.3), which exhibits a greater affinity for interacting with the buccal epithelium compared to the Tween^®^ 80 (HLB: 15) used in this study, potentially generating a more potent permeation-enhancing effect. However, the limited colloidal stability of LCMs (lipid-core micelles) [78], driven by the rapid kinetics of their polymeric constituents and their interactions with the surrounding medium and neighboring micelles, makes them less suitable when predictable, time-dependent permeation is required.

Likewise, the ability of this formulation to achieve values comparable to those reported in studies employing HPMC films associated with NLCs [79] and chitosan films combined with a polymeric nanoparticle [80] is noteworthy. This finding highlights the effectiveness of the current approach in attaining equivalent outcomes without the need to rely on the mucoadhesive properties provided by such systems [81], which typically entail prolonged residence and contact times and lead to a significant modification of the drug concentration gradient at the site of application.

## 5. Conclusions

Angiotensin II-associated NLCs were developed as a model buccal formulation for a hydrophilic peptide. The nanoparticles exhibited nanometric size, moderate polydispersity, and association efficiency above 80%, and remained colloidally stable at 4 °C over 28 days. In ex vivo Franz cell experiments using porcine buccal mucosa, Ang II-associated NLCs yielded measurable permeation under the tested conditions, whereas free Ang II was not detected in the receptor compartment. Importantly, this work establishes a controlled, quantitative ex vivo buccal transport comparison of a hydrophilic peptide model delivered as NLC-associated versus free peptide under matched Franz cell conditions. These findings should be interpreted within the limits of a proof-of-concept ex vivo study, as direct TEM characterization and full mass-balance recovery were not performed.

## Figures and Tables

**Table 1:** Summary of characterization of Free NLC and NLC loaded with 5.8 (NLC-Ang II) (*n* = 6) and 11.6 mg (NLC-Ang IIc) (*n* = 3) of Angiotensin II in terms of hydrodynamic diameter (HD) (nm ± SD), polydispersity index (PdI) (±SD), zeta potential (ZP) (mV ± SD) for all nanoparticles, association efficiency (AE) (±SD) and drug loading (DL) (±SD) in the case of those loaded with the peptide under study.

	HD (nm)	PDI	ZP (mV)	AE (%)	DL (%)
Free NLC	72.10 ± 8.91	0.261 ± 0.016	−6.198 ± 1.095	-	-
NLC-Ang II	83.73 ± 0.7	0.219 ± 0.010	−3.745 ± 0.401	87.29 ± 0.34	0.484 ± 0.003
NLC-Ang IIc	91.52 ± 0.19	0.263 ± 0.009	−2.893 ± 0.418	83.61 ± 0.06	0.907 ± 0.001

**Table 2:** Hydrodynamic diameter (average and SD) of empty NLCs and NLC-Ang II during the 28-day stability study.

Days	Empty NLC						NLC-Ang II					
	4 °C		25 °C		37 °C		4 °C		25 °C		37 °C	
	HD (nm)	SD (nm)	HD (nm)	SD (nm)	HD (nm)	SD (nm)	HD (nm)	SD (nm)	HD (nm)	SD (nm)	HD (nm)	SD (nm)
0	72.11	8.91	72.11	8.91	72.11	8.91	83.74	0.70	83.74	0.70	83.74	0.70
1	71.55	6.86	73.79	8.35	74.19	7.58	84.88	0.93	85.85	1.21	87.67	1.45
2	72.87	7.09	74.16	9.05	79.83	10.05	84.84	0.43	86.73	0.24	87.87	0.23
3	72.96	7.17	74.12	7.92	76.05	6.67	87.82	0.69	88.17	1.26	90.55	0.59
14	75.63	6.99	80.78	7.73	102.6	3.22	89.48	0.71	91.45	1.01	111.73	1.05
28	78.71	7.51	80.44	8.29	147.9	20.79	93.23	0.92	95.13	0.57	135.87	0.67

## Data Availability

The original contributions presented in this study are included in the article/Supplementary Material. Further inquiries can be directed towards the corresponding author.

## Supplementary Materials

The following supporting information can be downloaded at https://www.mdpi.com/article/10.3390/pharmaceutics18040416/s1, Figure S1. Cumulative in vitro release profiles of NLC associated with Angiotensin II (NLC-Ang II), performed at 37 °C, in phosphate buffer (pH 6.8) under the selected in vitro release conditions.

## Abbreviations

The following abbreviations are used in this manuscript:

RAS	Renin–angiotensin system
AT1R	Angiotensin II type-1 receptor
AT2R	Angiotensin II type-2 receptor
NLC	Nanostructured lipid carrier
Ang II	Angiotensin II
LOD	Limit of Detection
LOQ	Limit of Quantification
PDI	Polydispersity index
HD	Hydrodynamic diameter
DLS	Dynamic light scattering
ZP	Zeta potential
NLC-Ang II	Nanostructured lipid carrier associated with Ang II
NLC-Ang IIc	Nanostructured lipid carrier associated with concentrated Ang II
AE	Association efficiency
DL	Drug loading
PBS	Phosphate-buffered saline
FD-20	Fluorescein isothiocyanate–dextran 20 kDa
Jss	Steady-state flux
Papp	Apparent permeability coefficient
SD	Standard deviation
f_2_	Similarity factor
DLVO	Derjaguin-Landau-Verwey-Overbeek
DSC	Differential scanning calorimetry
SEDDS	Self-Emulsifying Drug Delivery Systems
LCM	Lipid-core micelles
HLB	Hydrophilic-Lipophilic Balance
HPMC	Hydroxypropylmethylcellulose
