# Supplementary material for: Ex Vivo Buccal Permeability of Nanostructured Lipid Carriers (NLCs) Associated with a Peptide Drug Model
Source: Pharmaceutics. 2026 Mar 29;18(4):416. doi: 10.3390/pharmaceutics18040416 (PMC13118935; doi:10.3390/pharmaceutics18040416)
Supplement: Supplementary file 1 [file pharmaceutics-18-00416-s001.zip › pharmaceutics-4202350-supplementary.pdf]

Supplementary Materials: Ex Vivo Buccal Permeability of Nanostructured Lipid Carriers (NLCs) Associated with a Peptide Drug Model

Sebastián Vargas-Valderrama and Javier O. Morales

In Vitro Release Profile of Ang II from Nanostructured Lipid Carriers (NLCs)

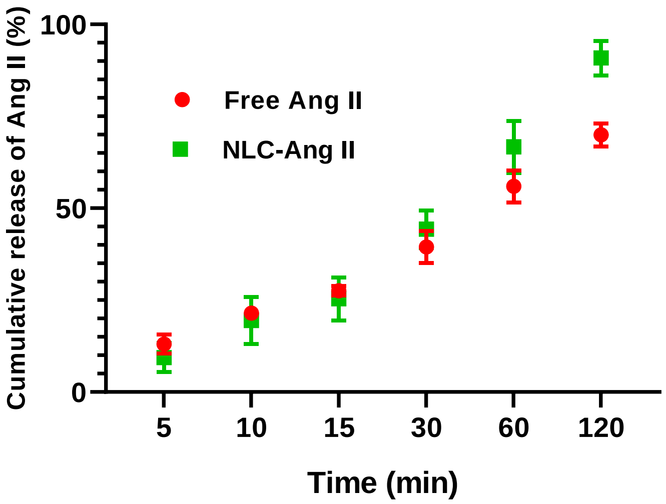

Figure S1. Cumulative in vitro release profiles of NLC associated with Angiotensin II (NLC-Ang II), performed at 37 °C, in phosphate buffer (pH 6.8) under the selected in vitro release conditions.

Release studies were conducted using dialysis bags under simulated physiological conditions (phosphate buffer, pH 6.8, 37 °C) for 2 h. PBS pH 6.8 was used as a simplified comparative release medium to assess relative release behavior under controlled conditions; it was not intended to fully reproduce the complete composition of saliva. Two experimental setups were compared: free Ang II and Ang II-loaded NLCs (NLC-Ang II). The peptide displayed considerable release from the NLCs, with over 25% (25.35 ± 5.87%) released at 15 min and more than 60% (66.74 ± 7.08%) after 1 h. To compare the release profiles of free Ang II and Ang II-loaded NLCs, the similarity factor (f2) was calculated, yielding f2 = 73.03. Since this value exceeds the acceptance threshold of 50, a high similarity was confirmed. Given that the release profile of Ang II from NLCs did not significantly differ from that of free Ang II, further kinetic modeling of the release process was deemed unnecessary.
